# Multilocular Cystic Renal Cell Carcinoma or Cystic Nephroma?

**DOI:** 10.1155/2016/5304324

**Published:** 2016-12-15

**Authors:** Adolfo González-Serrano, Roberto Cortez-Betancourt, Alejandro Alías-Melgar, Pedro Jair Botello-Gómez, Emilio Ramírez-Garduño, Eric Iván Trujillo-Vázquez, Yosimart Torres-Santos, José Antonio Mata-Martínez, Fernando Carreño- de la Rosa

**Affiliations:** Department of Urology, Centro Médico Nacional 20 de Noviembre, Mexico City, Mexico

## Abstract

The incidence of Multilocular cystic renal cell carcinoma (MCRCC) in literature is very low and confounding MCRCC with cystic nephroma (CN) is even more unusual. The aim of this report is to present a case of MCRCC and emphasize the importance of the preoperative radiologic evaluation and immunohistochemical staining confirmation to obtain an accurate diagnosis. A 73-year-old woman presented with a history of 4-month right flank pain. CT showed a Bosniak type III renal mass. After laparoscopic partial nephrectomy the initial report was cystic nephroma. Immunohistochemical staining was performed being positive for Epithelial Membrane Antigen thus changing the diagnosis to MCRCC. Multilocular cystic renal cell carcinoma cannot reliably be distinguished from cystic nephroma neither by physical examination nor by radiologic evaluation; immunohistochemical staining assay is useful to differentiate between these conditions allowing an accurate diagnosis and proper follow-up.

## 1. Introduction

Multilocular cystic renal cell carcinoma (MCRCC) was classified as a different subtype of renal cell carcinoma in 2004 by the World Health Organization [1].

At the 2012 International Society of Urological Pathology (ISUP) consensus meeting on adult renal neoplasia, the ISUP has designated the new term of “Multilocular cystic clear cell renal cell neoplasm of low malignant potential” due to the oftentimes reported nonaggressive behavior of MCRCC [2].

The 2016 WHO Classification of Tumors of the Urinary System and Male Genital Organs includes this new term and defines it as tumors composed entirely of numerous cysts, lined by a single layer of tumor cells with abundant cytoplasm with low-grade tumor cells. Cells displaying nuclear grade 2 are also acceptable in the diagnosis of MCRCC [3].

The incidence of MCRCC in literature is very low, reporting an incidence of 1-2% of MCRCC among renal cell carcinomas [4].

In spite of its low incidence and difficulties in accurate diagnosis, confounding MCRCC with cystic nephroma (CN) is even more unusual; in a PubMed search we found only two papers reporting this issue [5, 6].

The aim of this report is to present a case of MCRCC and emphasize the importance of the preoperative radiologic evaluation and immunohistochemical staining confirmation to obtain an accurate diagnosis.

## 2. Case Presentation

A 73-year-old woman presented with a history of 4 months of intermittent mild right flank pain. There was no relevant previous medical history and no family history of neoplasms. The patient had no significant weight loss, no anorexia, no fever, no hypertension, no urinary tract infections, no hematuria, and no gastrointestinal symptoms. Physical examination revealed slight tenderness in right flank palpation, with no palpable masses or peritoneal reaction in the abdomen palpation.

Laboratory findings from routine blood tests (hemoglobin, white cell count, platelets, creatinine, C-reactive protein, liver function test, and coagulation) and urinalysis were normal.

**Figure 1:**
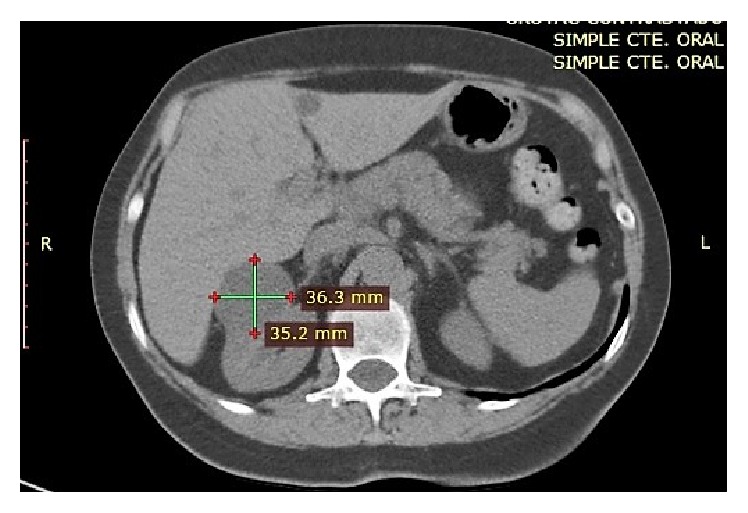
Noncontrast CT scan: water density renal mass, compatible with a simple renal cyst.

An abdominopelvic contrast-enhanced tomography was performed. Without contrast the CT revealed a well-defined limit, water density mass (15–17 HU) (Figure 1).

**Figure 2:**
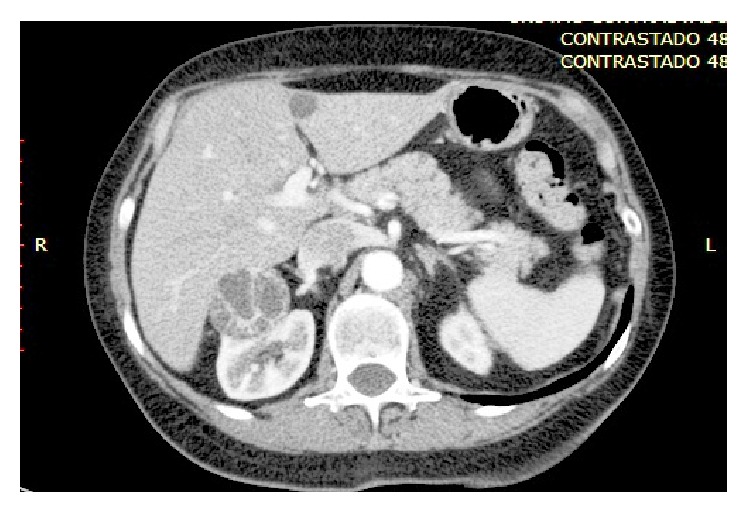
Contrast-enhanced CT scan (corticomedullary phase): complex cyst compatible with a Bosniak III cystic renal mass.

After contrast administration the CT demonstrated the multiloculated morphology of the renal mass with multiple cysts separated by multiple thick and irregular septa showing a 20 HU enhancement. No intrathoracic and abdominal lymphadenopathy was reported. These findings were consistent with a Bosniak type III lesion (Figure 2).

## 3. Treatment and Outcome

A laparoscopic partial nephrectomy without ischemia was performed and no complications were present during or after surgery.

Macroscopic examination of the surgical specimen showed a 4 × 3 × 2 cm pink, roughed, renitent, round mass with hemorrhagic content and multiple septa.

**Figure 3:**
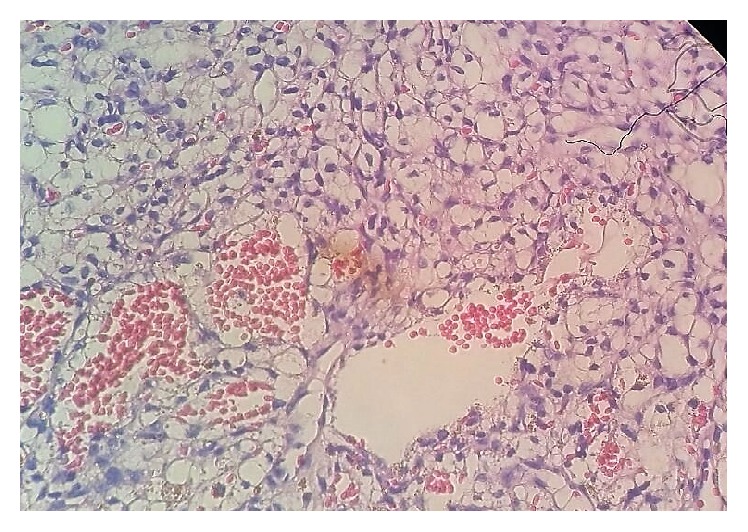
Photomicrograph histological section revealing multiple cysts and fibrous septa.

The microscopic evaluation revealed neoplasia with extensive areas of cystic degeneration and septa (Figure 3).

In a 10x view there were observed round clear cells with clear cytoplasm, compatible with CCR but initial diagnosis of cystic nephroma was made by first pathological evaluation.

**Figure 4:**
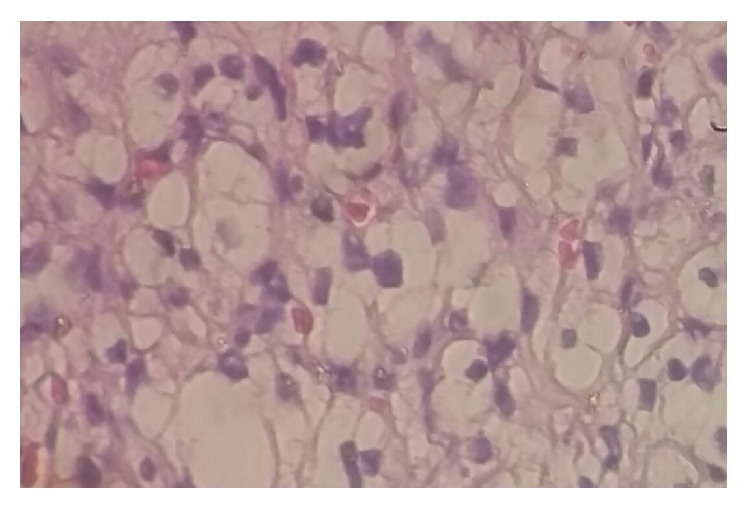
Photomicrograph at 10x showing clear round cells without nucleoli.

According to another pathologist assessment, these clear cells showed low Fuhrman nuclear grade and there was no ovarian-like stroma; in his opinion immunohistochemistry was needed to perform an accurate diagnosis (Figure 4).

Because of the mass behavior in CT and according to microscopic findings we decided to perform immunohistochemical staining which resulted negative to estrogen and progesterone receptors and CD10; positivity for Epithelial Membrane Antigen (EMA) was demonstrated.

With these findings, the definitive pathological diagnosis changed from cystic nephroma to MCRCC with a Fuhrman grade of 1, with surgical margins negative for neoplasia.

## 4. Differential Diagnosis

Bosniak type III lesions are undetermined in their malignant potential. Malignancy is found in over 50% of Bosniak type III lesions. Such tumors like renal cell carcinoma (RCC), cystic RCC, tubulocystic carcinoma or clear cell papillary RCC can present with cystic, necrotic or hemorrhagic changes, and nuclear grade 2 of Fuhrman in two-thirds of these tumors (61%) [7].

Benign renal masses can also be part of the differential diagnosis of MCRCC. This includes mixed epithelial and stromal tumors of the kidney, cystic nephroma, Multilocular cysts, and renal abscess [8].

## 5. Discussion

The diagnostic evaluation of patients with MCRCC is troublesome before surgery due to the nonspecific radiological findings of this pathology. Most of these renal masses are classified according to the Bosniak cyst classification system in an attempt to predict the malignant potential of these lesions [9].

According to these findings, some studies have tried to differentiate between MCRCC of other cystic RCC. You and coll. proposed a diagnostic algorithm using a Bosniak classification and Hounsfield units to predict the probability of finding MCRCC versus other types of cystic RCC. They used a cut point of ≥38 HU in the corticomedullary phase and found that the HU during this phase was significantly higher in other types of RCC, having a 83% sensitivity and 80% specificity with an area under the ROC curve of 0.886 (95% CI 0.808–0.963; *p* < 0.001) for predicting other RCC [10].

In our case the reported HU in the corticomedullary phase were 37 HU, thus supporting the fact of facing a MCRCC.

More specifically, one study by Zhao and coll. [11] tried to improve the accuracy of preoperative diagnosis between CN and MCRCC. They observed that shallow lobulation, protruding to the renal sinus, thin walls, and partitions without nodules, favored CN and net growth in the cortical and nephrographic phase, thick walls, nodules, and higher enhancement after contrast media administration indicated a higher possibility of MCRCC. All differences were statistically significantly different (*p* < 0.05).

Histologically, there are different features that could help distinguish cystic nephroma from MCRCC. In CN, there are focally distributed clear cells in the surface of the septa, hobnail epithelium, ovarian-like stroma, and mature tubules in the septa, whereas evident solid areas in cystic mass or extensile nodules of clear cells favor MCRCC [12, 13].

Definitive diagnosis is made immunohistochemically. As all cystic renal tumors have epithelial component, it is important to differentiate by other methods rather than microscopy between the malignant components of these epithelial cells.

A few studies have performed immunohistochemical analyses to identify malignant cells from benign epithelial cells. One of them by Zhang et al. [14] compared 19 cases of MCRCC versus other cystic kidney lesions and 22 benign simple cortical cysts as controls. They observed that the cysts lined epithelial cells and the clear tumor cell clusters were positive for epithelium markers like CKpan (19/19), EMA (16/19) and CK7 (15/19), CA-IX (17/19) and PAX8 (15/19), and a low percentage staining for CD10 (7/19).

Another study assessed the immunohistochemical staining characteristics, of MRCC versus control cases, showing the following results, respectively: CD10 (63% versus 96%), CK7 (92% versus 38%), *α*-methylacyl-CoA-racemase (21% versus 67%), vimentin (58% versus 33%), estrogen receptor (8% versus 8%), CAM 5.2 (100%, 96%), EMA, CA-IX, PAX-2 (100%), and progesterone receptor (0%) [15].

As reported by Turbiner and coll. [16], a detailed pathologic analysis of 22 CN revealed that ovarian-like stroma, estrogen and progesterone receptors, CD10 positivity, calretinin, and inhibin support the diagnosis of CN; in our case all these markers were negative and this information was useful to discard the initial diagnosis of CN.

Therefore we can assume that useful immunohistochemical staining for EMA, CK7, and CA-IX may be helpful in establishing a more accurate diagnosis and differentiating other cystic lesions from MCRCC as we saw in our case.

Concerning the best modality of treatment for these patients, literature is still controversial but there may be some preference to treat MCRCC by partial nephrectomy due to its low aggressive potential.

In one of the larger series reporting treatment and outcomes, they treated 76 patients with MCRCC; 18 underwent radical open nephrectomy, 18 laparoscopic radical nephrectomy, 22 open partial nephrectomy, and 18 laparoscopic partial nephrectomy. 66 patients were followed up from 3 to 113 months (median, 52 months); at the last follow-up date, all patients were alive, with exception for 1 patient who died of rectal cancer, and no patient showed signs of metastasis or local recurrence [17].

In a series of 2679 with RCC treated in a single center they found 67 cases of MCRCC. 19 patients were treated by open radical nephrectomy, 12 open partial nephrectomy, 9 laparoscopic radical nephrectomy, and 20 laparoscopic partial nephrectomy. 47 patients were followed up for a mean of 42 months (mean 6–84) and they found no evidence of recurrence or metastasis. Four patients died of non-cancer-related causes [18].

As in these studies, we decided to perform a laparoscopic partial nephrectomy showing satisfactory clinical and oncological results after a follow-up of 10 months.

## 6. Conclusion 

Multilocular cystic renal cell carcinoma cannot reliably be distinguished from cystic nephroma neither by physical examination nor by radiologic evaluation; immunohistochemical staining assay is useful to differentiate between these conditions allowing an accurate diagnosis and proper follow-up.

Accurate diagnosis is always important in a cancer context even the low malignant potential of this tumors.
